# A novel compound 6D‐offset simulating phantom and quality assurance program for stereotactic image‐guided radiation therapy system

**DOI:** 10.1120/jacmp.v14i6.4297

**Published:** 2014-11-04

**Authors:** Dennis Yuen‐Kan Ngar, Michael Lok‐Man Cheung, Michael Koon‐Ming Kam, Wai‐Sang Poon, Anthony Tak‐Cheung Chan

**Affiliations:** ^1^ Department of Surgery The Chinese University of Hong Kong Shatin Hong Kong; ^2^ Department of Clinical Oncology Prince of Wales Hospital Shatin Hong Kong; ^3^ Department of Clinical Oncology The Chinese University of Hong Kong Shatin Hong Kong; ^4^ The Hong Kong Cancer Institute State Key Laboratory in Oncology in South China The Chinese University of Hong Kong Shatin Hong Kong

**Keywords:** IGRT, ExacTrac, quality assurance, 6D compound offset, 6D phantom

## Abstract

A comprehensive quality assurance (QA) device cum program was developed for the commissioning and routine testing of the 6D IGRT systems. In this article, both the new QA system and the BrainLAB IGRT system which was added onto a Varian Clinac were evaluated. A novel compound 6D‐offset simulating phantom was designed and fabricated in the Prince of Wales Hospital (PWH), Hong Kong. The QA program generated random compound 6D‐offset values. The 6D phantom was simply set up and shifted accordingly. The BrainLAB ExacTrac X‐ray IGRT system detected the offsets and then corrected the phantom position automatically through the robotic couch. Routine QA works facilitated data analyses of the detection errors, the correction errors, and the correlations. Fifty sets of data acquired in 2011 in PWH were thoroughly analyzed. The 6D component detection errors and correction errors of the IGRT system were all within ±1mm and ±1° individually. Translational and rotational scalar resultant errors were found to be 0.50±0.27mmand0.54±0.23°, respectively. Most individual component errors were shown to be independent of their original offset values. The system characteristics were locally established. The BrainLAB 6D IGRT system added onto a regular linac is sufficiently precise for stereotactic RT This new QA methodology is competent to assure the IGRT system overall integrity. Annual grand analyses are recommended to check local system consistency and for external cross‐comparison. The target expansion policy of 1.5 mm 3D margin from CTV to PTV is confirmed for this IGRT system currently in PWH.

PACS numbers: 87.53.Ly, 87.55.Gh, 87.55.Qr, 87.56.Fc

## I. INTRODUCTION

### A. Image‐guided radiation therapy (IGRT)

Precision or stereotactic image‐guided radiosurgery and radiotherapy have become common practice in radiation oncology. This is the technique developed to achieve high accuracy in target positioning without the stereotactic frame. Without IGRT or the frame, patients were set up according to external surface marks such as skin marks and shell marks. These marks are loose and inconsistent. IGRT treatment system is able to detect six degree of freedom or six‐dimensional (6D) patient positioning error by internal structure imaging with computer algorithm,^(1)^ and then to correct the compound errors by moving the patient on the robotic treatment couch with servo‐tracking.^(2)^ As the IGRT technology turned mature, even the conventional stereotactic frame could be replaced by it.^(^ ^3^ ^,^ ^4^ ^)^

**Figure 1:**
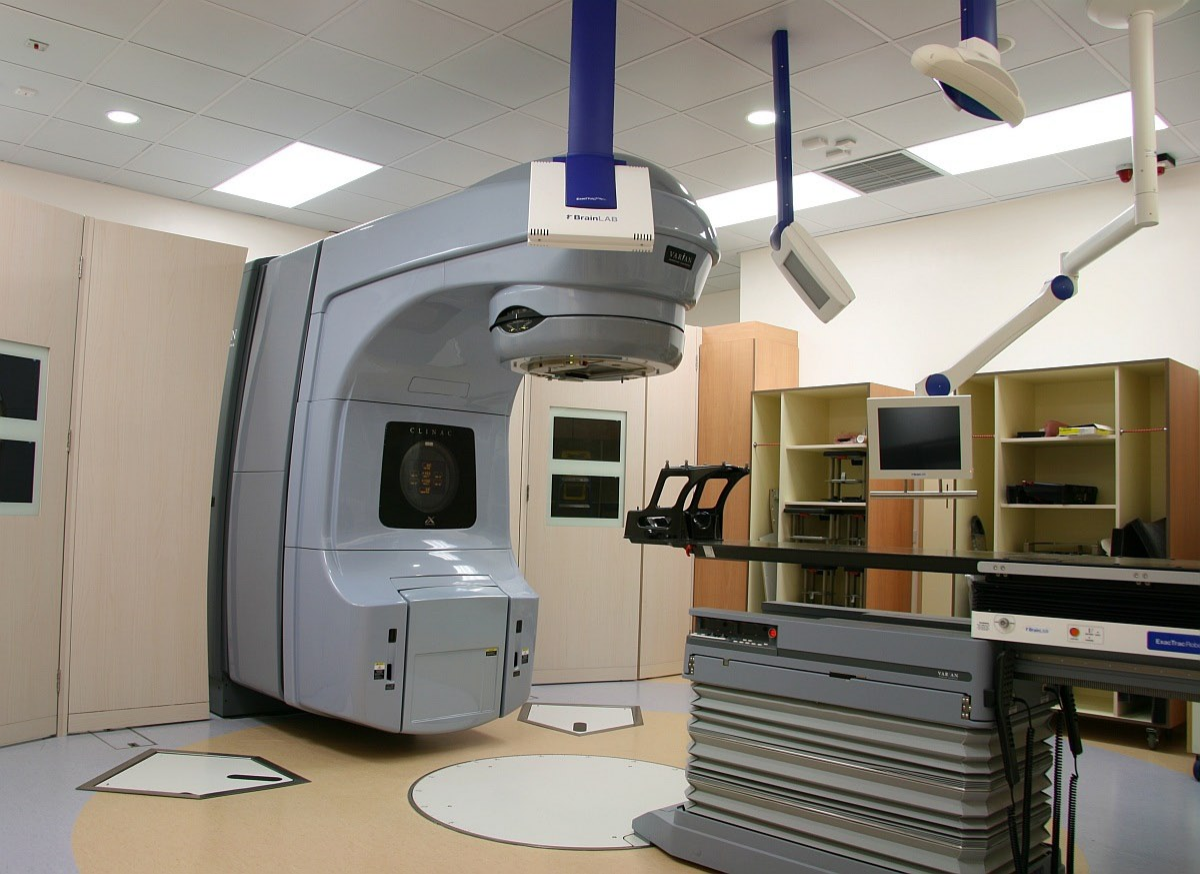
The IGRT suite comprises a Varian iX Clinac and an add‐on BrainLAB ExacTrac stereoscopic X‐ray cum 6D robotic couch system in PWH.

Theoretically, two well‐separated 2D orthogonal images of the target are required and sufficient to detect all the 6D positional errors when the real‐time images are compared with the reference ones. IGRT is applied to RT cases requiring high precision of patient setup. Typical examples are the head & neck cases and the spine cases. Real‐time images for IGRT can be obtained by two fixed oblique stereoscopic X‐ray with flat‐panel receivers, or by the built‐in On‐Board Imager moving isocentrically. The latter can even form 3D images through the cone‐beam CT function. It was shown that the stereoscopic X‐ray system and the CBCT system exhibited similar accuracy of IGRT function.^(5)^ There are at least two 6D IGRT robotic patient setup products available on the market, namely the BrainLAB ExacTrac (BrainLAB, Feldkirchen, Germany) and the Elekta HexaPOD (Elekta, Stockholm, Sweden). The combined imaging and robotic IGRT system can be acquired separately and added onto an existing linac (Fig. 1). The CyberKnife system (Accuray Inc., Sunnyvale, CA) also incorporates a 6D patient setup device.

### B. Six‐dimensional position error

Every space‐occupying object shall have six degrees of freedom: three translational and three rotational. A real patient therefore shall have 6D setup error on the RT treatment couch, generally. The real patient 6D errors shall be compound and random. The linac couch 6D displacements are vertical, longitudinal, lateral, yaw, roll and pitch. In external‐beam radiation oncology, the three rotations of yaw, roll, and pitch are measured about the planned center of the treatment target, or the isocenter of the linac.

### C. The standard of accuracy

The scope of performance of a linac‐based stereotactic system was established by AAPM Report No.54.^(6)^ The early stereotactic radiosurgery system was frame‐based, with four screws securing the frame to the skull of the patient. The 3D spatial accuracy required was within 1 mm in three translational dimensions.

Rotational setup error can be risky to the patient when the target or OAR, or both, are elongated in shape. The error would cause partial missing of the target or overdosing the normal organ, or both, even when the translational setup is accurate. A typical example of this is the treatment setup for the spinal lesions, for which the relevant rotational setup errors should not exceed 2°.^(7)^ Considering a typical target with an extent of 10 cm, a rotation of 1° about the center would cause the whole periphery of the mass to shift about 1 mm tangentially (50mm×sin1°). Therefore, by this argument, the rotational accuracy required should be within 1° in the three senses of rotation to match with the translational standard.

BrainLAB ExacTrac X‐ray system and the Novalis Linac system (BrainLAB AG) with built‐in IGRT function claimed submillimeter accuracy and the static target positioning error of which had been reviewed over the past ten years.^(^ ^3^ ^,^ ^4^ ^,^ ^8^ ^,^ ^9^ ^,^ ^10^ ^,^ ^11^ ^,^ ^12^ ^,^ ^13^ ^,^ ^14^ ^,^ ^15^ ^)^ One of the purposes of this paper is to investigate independently the accuracy of an add‐on BrainLAB ExacTrac IGRT and Robotic 6D patient positioning system with the Varian iX linear accelerator installed in our center (Fig. 1). The hypothesis was that the system could also achieve 1 mm accuracy in each of the three translational dimensions and 1° accuracy in each of the three rotational dimensions simultaneously. Another purpose of this study is to develop the required novel comprehensive QA system.

### D. Quality assurance (QA)

The versatile stereotactic IGRT system deserves the support of a comprehensive, meticulous, and powerful QA system before the system is routinely claimed precise and reliable.

#### D.1 Internal calibration

**Figure 2:**
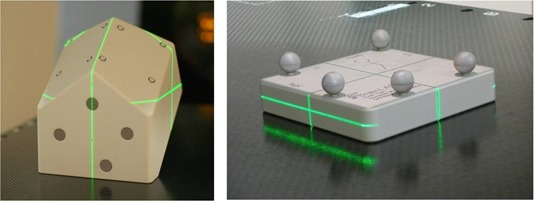
The IGRT calibration phantoms for the stereoscopic X‐ray imaging units (left) and for the infrared tracking units (right). They are factory products supplied by BrainLAB. Both are not QA devices.

Linac peripheral equipments like the IGRT system must be calibrated and have the QA done regularly. In fact, the ExacTrac IGRT system is sensitive to spatial aberrations of the hanging flat panels or the infrared cameras (Fig. 1). System calibration is mandatory, yet the process and the results are only internal (Fig. 2). It doesn't serve to assure the system quality directly and quantitatively.

#### D.2 Winston‐Lutz test

IGRT calibration is based on the laser system for patient positioning of the linac. It is assumed that the laser system is congruent to the linac isocenter. A good Winston‐Lutz test result^(16)^ is prerequisite to the calibration.

#### D.3 Daily quick check

**Figure 3:**
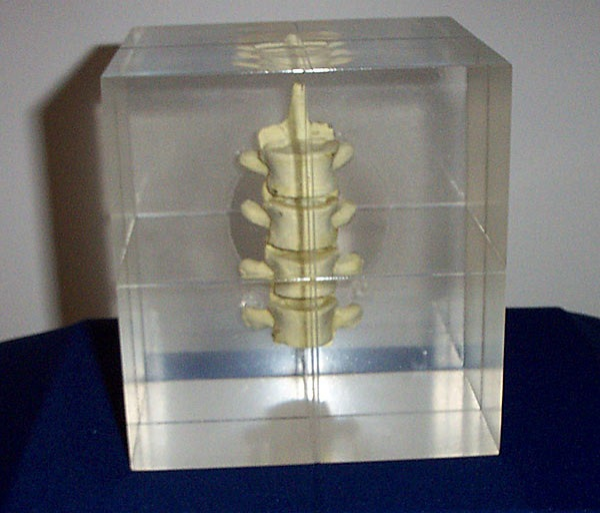
The Alderson IGRT QA Phantom.

Gadgets for daily quick check of the IGRT system are available; one example is the Alderson IGRT QA Phantom (Radiology Support Devices Inc., Long Beach, CA) (Fig. 3). The rigid block can be set by hand and shifted from the reference position on the couch. As the usual images are taken, offsets are detected and finally the block is automatically brought back to the reference position, fulfilling the purpose of an IGRT quick check. However, as the initial compound 6D shift is not accurately known, detection performance evaluation becomes impossible. Postcorrection verification is also illogical by the quick check only since it is obtained from the second detection. Care must also be taken to make sure that the original offsets do not exceed the system's limits, otherwise the quick check would fail.

#### D.4 Full meticulous phantom QA

It seems clear that a full, meticulous, and routine IGRT QA system is absolutely necessary. Quality assurance results shall be quantitative, objective, and be comprehensive as much as possible. In this study, a new QA system was developed to facilitate the task.

## II. MATERIALS AND METHODS

### A. The BrainLAB Exactrac X‐ray Cum Robotic Couch IGRT System

The idealized working theory of the stereoscopic X‐ray cum robotic couch IGRT system is given in Table 1. It is obvious that from CT planning, stereoscopic imaging, DRR generation, image fusion, 6D detection, offset calculation, markers tracking, 6D couch correction, and finally to phantom alignment, the processes were interrelated and the proof of precision was absolutely nontrivial. A practical end‐to‐end QA system on this IGRT performance is essential.

**Table 1:** The idealized workflow and theory of the ExacTrac IGRT system. The flow runs from the top row to the bottom. Detection is based on the X‐ray system, while correction is based on the Infrared system

	*Phantom 6D Vector*	*Markers 6D Vector*	*Remarks*
General	P	M=P+K	Markers are attached on the Rigid Phantom, K is a constant
6D Neutral N	$P_{0}=N$	$M_{0}=N+K$	N refers to the reference planning CT image set
6D Offset E	$P_{1}=N+E$	$M_{1}=N+K+E$	E is random and compound Phantom is shifted
6D Detection on Phantom Image	E		Markers are not detectable
6D Correction by Markers Tracking		$M_{2}=M_{1}-E=N+K=M_{0}$	Phantom is not trackable
Final 6D Config.	$P_{2}=M_{2}-K=P_{0}$		Phantom is brought back to the reference position

### B. The Compound 6D QA Phantom and Ancillary Gadgets

A novel compound 6D‐offset simulating phantom cum quality assurance program for precision image‐guided radiosurgery and radiotherapy was developed for the QA purpose. The phantom (patent pending) was developed as a prototype in the Medical Physics Unit in PWH. This design and technology allows the user to carry out QA works of an IGRT system comprehensively and simply in one sequence with a random, compound 6D methodology.

#### B.1 The phantom body for compound offsets

**Figure 4:**
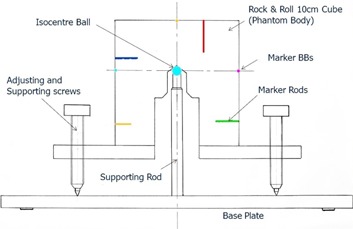
A schematic (not engineering) drawing of the 6D QA phantom (patent pending) showing the essential parts that can fix the phantom body in any compound 6D offsets for the QA purposes.

**Figure 5:**
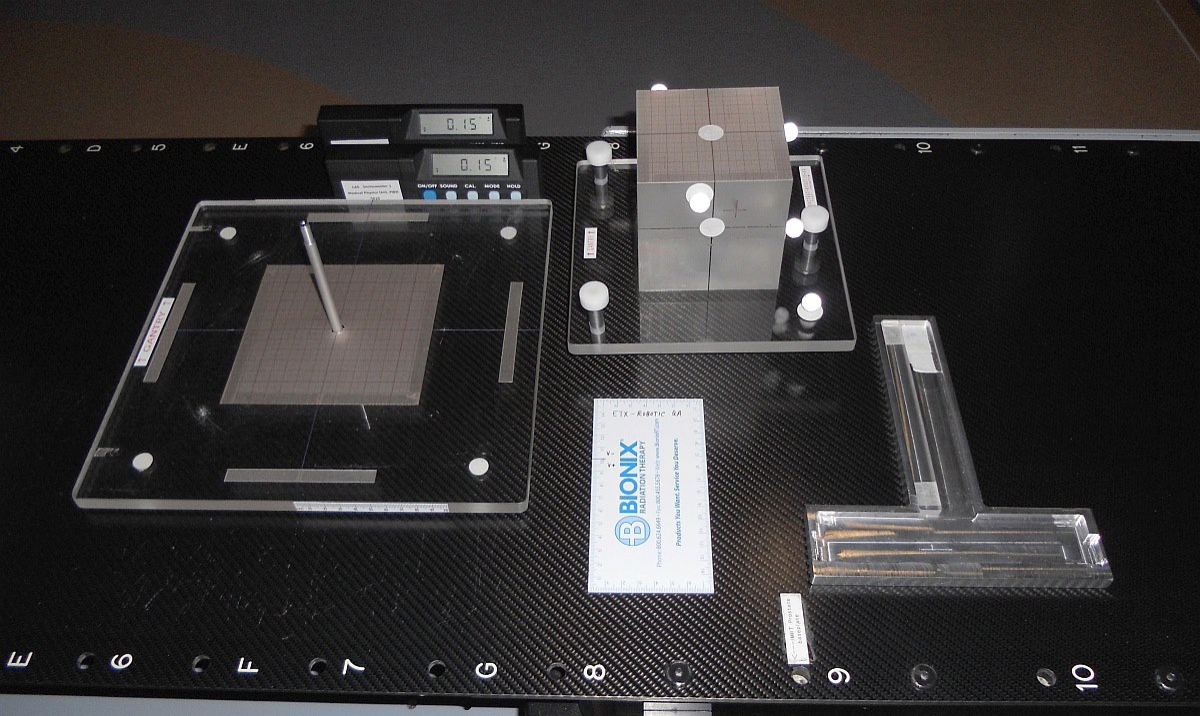
The complete set of ancillary gadgets of the 6D QA phantom including the base plate, the phantom body, the two inclinometers with their T‐tray, and the measuring devices.

The phantom body is basically a 10 cm‐sided precisely machined Perspex cube (Fig. 4). There is an opening or vault at the bottom. The vault ends up at a center position like a socket. This essential position is termed the isocenter. A rigid, light, vertical rod with a steel ball end is supporting the phantom body at the isocenter. The whole phantom hence can rock and rotate about the supporting ball and rod, simulating the compound 3D rotations of yaw, roll, and pitch simultaneously about the isocenter. The rotations are adjusted and supported by three anchoring carbon fiber screws standing on the base plate. Generated rotations of roll and pitch are measured simultaneously by two calibrated electronic inclinometers with 0.1° resolution on a T‐tray (Fig. 5), which is placed on the top surface of the phantom. The yaw and the translational offsets are made with the aid of the infrared system and verified by the linac frame of measurement. Orthogonal cross‐lines are engraved accurately on the five faces of the cube, with the crosses aligning exactly with the isocenter. These engraved lines will match with the linac lasers and field cross‐hairs when the phantom is in neutral position, or after the completion of the robotic correction of the shifted 6D phantom. Metallic ball bearings and rods are embedded in the cube for radiological detection by the IGRT system. These ball and rod markers are for image matching or automatic fusion by the computer software. Silvery reflective balls are installed firmly on the phantom for the infrared cameras to monitor and to track the 6D robotic couch correction motion by servo control mechanism. According to BrainLAB, the configuration of the infrared balls on the target object is not required to be specific.

#### B.2 Neutral configuration and reference image set

**Figure 6:**
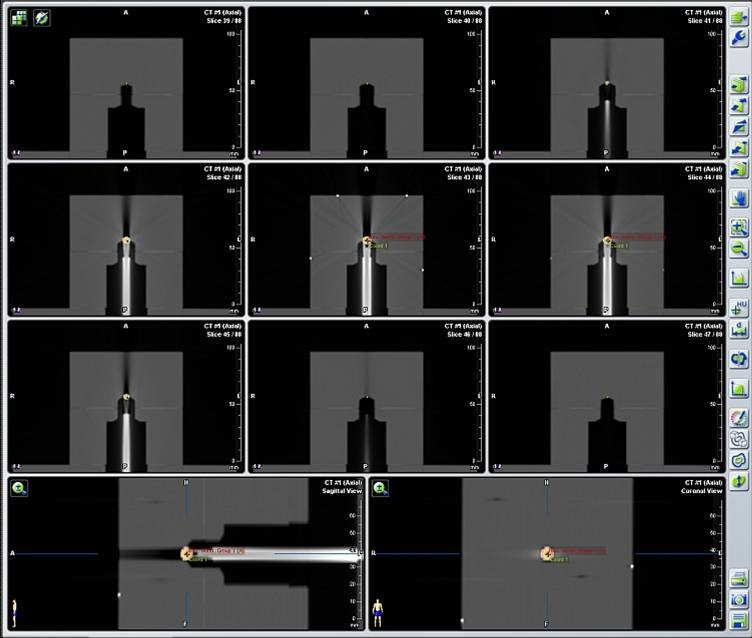
The planning CT images of the 6D phantom in neutral configuration (all zero offsets) showing the cross‐section and internal geometry of the phantom on the supporting rod. Note that there was image artifact above the metal ball due to the existence of the metal ball and the rod. These CT images constituted the reference image set for the QA program.

The standard reference neutral offset configuration was obtained by CT scanning of the phantom with zero offsets in six dimensions. The scanning parameters were 1.5 mm slice thickness in axial mode with field of view 350 mm. These are standard of a real patient with stereotactic head & neck treatment. The reference images (Fig. 6) were sent to the ExacTrac IGRT setup computer via the iPlan treatment planning computer. This reference CT image set of the 6D phantom will be stored in the IGRT computer for use in all the future QA applications.

#### B.3 Practical QA operation

**Figure 7:**
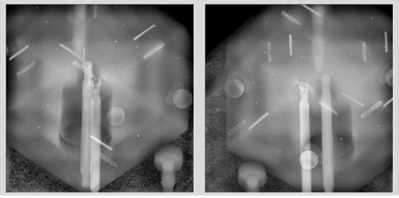
The 2D real‐time images; the 2D‐3D DRRs were overlaid, fused, and then the 6D offsets were calculated by the ExacTrac computer.

**Figure 8:**
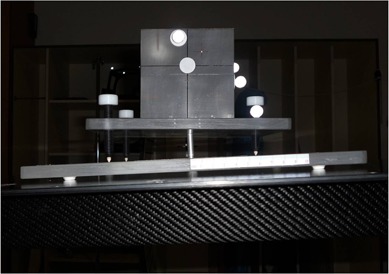
The 6D phantom was brought back to the reference configuration after precise robotic couch correction. The linac couch top was clearly dipped to compensate for this.

Two real‐time 2D stereoscopic images were taken with the offset phantom on the treatment couch. When the images were compared to the reference images digitally reconstructed (DRR) from the CT images, the 6D offsets were calculated by the system algorithm^(16)^ (Fig. 7). The detection performance was then evaluated. The system then proceeded to 6D robotic correction by allowing the automated couch motion (Fig. 8). By a second detection or verification, the robotic mechanical control performance or the correction error could also be evaluated. The QA works on the 6D phantom are very much similar to that of treating the real patient on the linac couch. Manual checking of the automatic software fusion of the DRR and the real‐time images is essential. Fusion ambiguities are always possible. All the markers on the phantom and the phantom body outline itself shall be carefully checked for congruence after the fusion. Masking the support rod and the leveling screws in Fig. 7 from taking part in the fusion is an essential step in the QA workflow.

### C. The Random 6D QA Program

**Figure 9:**
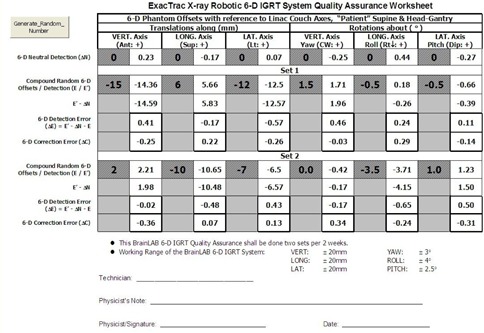
The sample ExacTrac X‐Ray robotic couch 6D IGRT system, and QA Excel worksheet with one set of neutral detection ΔN and two sets of 6D random offsets E. The two sets of 6D result values of ΔE and ΔC are shown. Note the sign conventions and the working ranges of the IGRT system being used.

**Figure 10:**
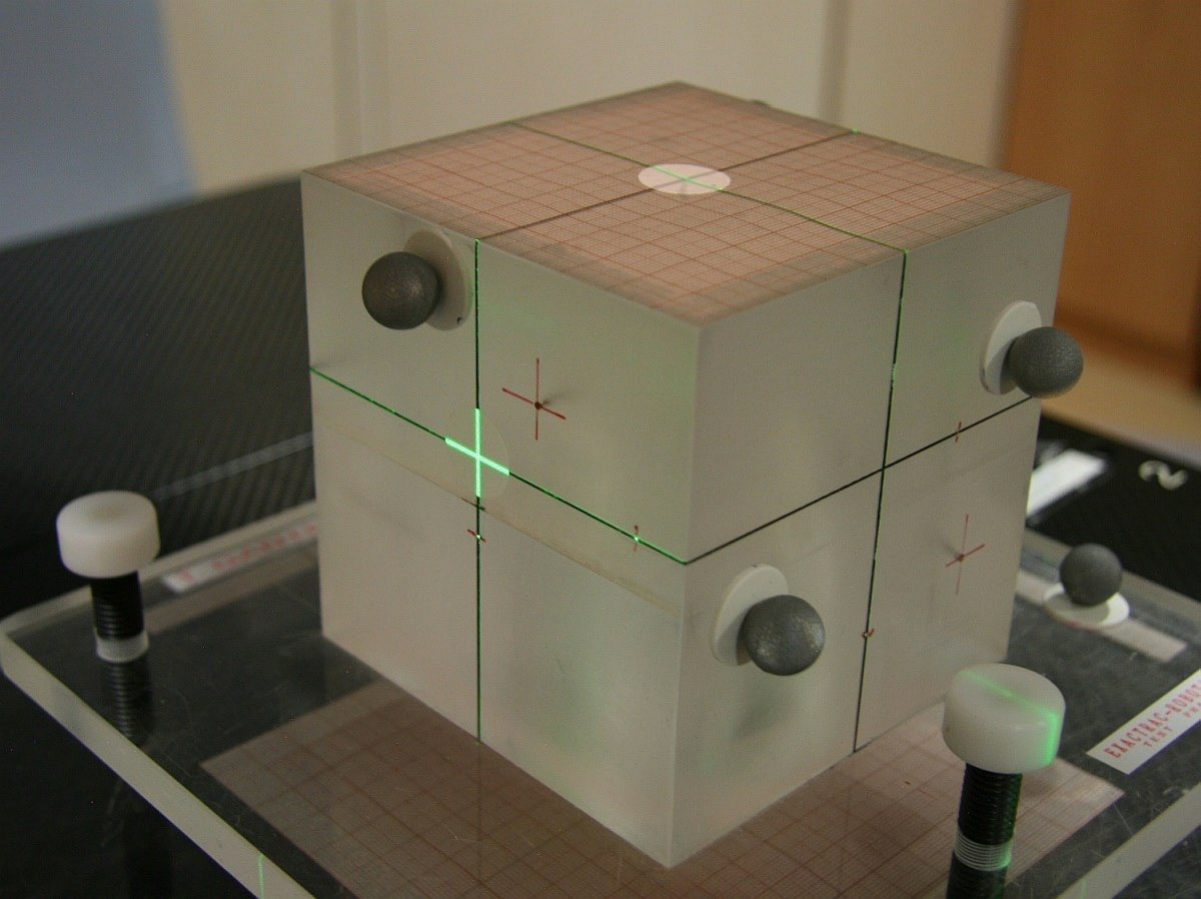
The 6D QA phantom in neutral configuration (all zero offsets) or after successful robotic correction, with the evidence of good alignments to the linac room lasers.

Natural offsets are 6D, random, and compound. To simulate them, two sets of random 6D compound offsets are generated each time on an Excel worksheet, as shown in Fig. 9. ΔN is the detection of the phantom in neutral configuration of the linac system. This is the small discrepancy between the CT images coordinate frame and the linac one. ΔN is consistent statistically and therefore should be subtracted from E', the 6D offset detection. Where E is the random offset, detection error is given by ΔE=E′−ΔN−E. ΔN will be discussed in detail in the Results section A.1 below. The working range of the IGRT robotic system was factory‐stated. The 6D random offsets E were generated by computer to fall evenly within the range (Fig. 9). The translational components of E were generated in 1 mm steps, while the rotational ones were multiples of 0.5° angles. ΔC was the correction error obtained by postcorrection detection. ΔN, E', and ΔE were ExacTrac readouts and were input directly to the QA worksheet. Figure 10 shows the phantom alignment with the room laser after the successful automated robotic correction for one set of 6D offset simulated. The QA program of two sets of random offsets at a time was done every two weeks. About 50 sets of data were obtained annually.

**Figure 11:**
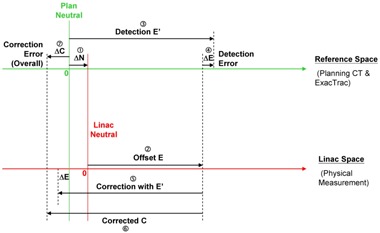
The generalized 1D illustration of ΔN, ΔE and ΔC in the IGRT QA system developed showing the mutual relationship among them. The circled numbers show the order of the QA procedures.

Without loss of generality, the 6D vectors ΔN, ΔE, and ΔC can be reduced to scalar 1D values for the easy, comprehensive illustration of them, as shown in Fig. 11. The circled numbers are showing the order of the QA procedures. The reference space and the linac space are placed side by side to show their mutual relationship. The opposite signs of ΔE and ΔC are well explained, and this will be discussed with the findings in the Results section B.3 below.

## III. RESULTS

### A. Numerical data and statistical analyses

According to the QA program and Fig. 9, 50 sets of compound random 6D offsets were generated, detected, and corrected on the 6D phantom and the IGRT system in one year, together with 25 sets of neutral configuration detection. The results were analyzed and presented in statistical parameters in Tables 2 and 3, where Resultant Translation is identical to Root Sum Square of (Vert, Long, Lat) and Resultant Rotation is identical to Root Sum Square of (Yaw, Roll, Pitch) by definition.

**Table 2:** The annual statistical analysis of 25 sets of neutral detection (all zero offsets) of the 6D phantom in PWH

		*Translational Components*			*Rotational Components*		
		*VERT*	*LONG*	*LAT*	*YAW*	*ROLL*	*PITCH*
N		25	25	25	25	25	25
	Max	0.62	0.19	0.87	0.67	−0.1	0.61
6D	Min	−0.46	−0.38	−0.36	−0.52	−0.96	−0.58
Neutral Detection	μ	0.08	−0.06	0.34^a^	0.08	−0.56^a^	−0.17^a^
ΔN	p(μ=0)	0.209	0.147	0.000	0.211	0.000	0.020
(mm|deg)	σ	0.26	0.18	0.34	0.29	0.18	0.29
	Median	0.05	−0.07	0.26	0.07	−0.57	−0.20

a The significant mean (with p < 0.05).

**Table 3:** The 2011 annual statistical analyses of 50 sets of 6D random compound offsets, detection errors, and correction errors and their distributions for the IGRT system in PWH. The linear regression figures (A, B) and the correlations (r) share the same p‐values correspondingly in the calculation

		*Translational Components*			*Rotational Components*			*Resultant*	
		*VERT*	*LONG*	*LAT*	*YAW*	*ROLL*	*PITCH*	*Trans*.	*Rot*.
System Limit (mm | deg)		±20	±20	±20	±3	±4	±2.5		
N		50	50	50	50	50	50	50	50
	Max	17	20	18	3	4	2.5	25.87	5.10
6D	Min	−20	−19	−20	−3	−4	−2.5	7.35	1.22
Random									
Offset E	μ	−1.04	1.28	−2.50	−0.18	0.67	0.02	17.11	3.10
(mm|deg)	σ	9.92	10.63	10.51	1.67	2.38	1.34	5.67	0.96
	Median	−1.5	3	−6	0	1.25	0.5	17.81	3.20
	Max	0.55	0.93	0.85	0.67	0.89	0.86	1.22	1.33
	Min	−0.97	−0.95	−0.83	−0.67	−0.97	−0.81	0.20	0.21
6D									
Detection	μ	−0.15^a^	0.06	0.01	−0.01	−0.11	0.12^a^	0.66	0.63
Error AE	p(μ=0)	0.012	0.283	0.827	0.855	0.057	0.047		
(mm|deg)	σ	0.39	0.41	0.41	0.36	0.40	0.40	0.28	0.28
	Median	−0.11	0.09	−0.04	−0.11	−0.19	0.09	0.62	0.62
	P(μ±2σ)	48/50	47/50	48/50	50/50	46/50	49/50	50/50	49/50
	Max	0.97	0.51	0.46	0.62	0.90	0.78	1.26	1.14
	Min	−0.98	−0.82	−0.46	−0.61	−0.68	−0.64	0.07	0.15
6D									
Correction	μ	0.10	−0.06	0.03	0.09^a^	0.05	−0.04	0.50	0.54
Error AC	p(μ=0)	0.115	0.150	0.339	0.039	0.340	0.408		
(mm|deg)	σ	0.44	0.27	0.22	0.31	0.36	0.33	0.27	0.23
	Median	0.10	0.00	0.06	0.09	0.06	0.00	0.47	0.49
	P(μ±2σ)	49/50	47/50	48/50	49/50	48/50	49/50	48/50	48/50
	ΔE to Offset	0.18	0.03	0.06	−0.37^a^	−0.29^a^	−0.42^a^	0.01	0.18
	p(r=0)	0.207	0.846	0.686	0.009	0.040	0.003	0.951	0.214
Correlation	ΔC to Offset	−0.15	−0.07	0.19	0.15	0.31^a^	0.54^a^	−0.15	0.14
r	p(r=0)	0.296	0.618	0.188	0.313	0.028	0.000	0.311	0.327
	ΔC to ΔE	−0.42^a^	−0.60^a^	−0.04	−0.31^a^	−0.64^a^	−0.60^a^	0.12	0.29^a^
	p(r=0)	0.003	0.000	0.771	0.029	0.000	0.000	0.398	0.043
	ΔE to Offset	−0.14	0.06	0.02	−0.02^a^	−0.08^a^	0.12^a^	0.65	0.46
Linear		0.01	0.00	0.00	−0.08^a^	−0.05^a^	−0.12^a^	0.00	0.05
Regression	ΔC to Offset	0.09	−0.05	0.04	0.10	0.02^a^	−0.04^a^	0.62	0.43
		−0.01	0.00	0.00	0.03	0.05^a^	0.13^a^	−0.01	0.03
Intercept A									
Slope B	ΔC to ΔE	0.03^a^	−0.03^a^	0.03	0.09^a^	−0.01^a^	0.02^a^	0.42	0.39^a^
		−0.47^a^	−0.40^a^	−0.02	−0.26^a^	−0.57^a^	−0.50^a^	0.12	0.23^a^

a The significant mean or correlation parameter (with p < 0.05).

#### A.1 Neutral detection ΔN

The 6D phantom was setup on the treatment couch with neutral configuration (all zero 6D components as Fig. 10). The orientation of the phantom agreed with the linac alignment system, the rulers, and the inclinometers. After taking the two stereoscopic images of the 6D phantom followed by software calculation, the 6D neutral detection ΔN against the DRR reference images was obtained. There were significant, systematic nonzero values of the mean of the 6D neutral detection ΔN (as shown in Table 2); they were all bounded within ±1mmand±1°. The ΔN may be due to the error of workmanship of the prototype 6D phantom, the error of the neutral setup of the phantom at the CT scanning, the error of aligning the isocenter at the computer planning stage, the intrinsic 6D offset of the center of the CT scanner, the intrinsic 6D error of the linac laser alignment system, or any combinations of these. The mean and standard deviation of the 25 data for each dimension were given by μ±σ in Table 2. Since the nonzero 6D Neutral offset seems to be inevitable practically, and the detected shift vector E’ had already included AN, the detection error ΔE shall include the subtraction of the corresponding component of AN.

#### A.2 Detection error AE

According to the QA program designed (Fig. 9), a set of random compound offset E was generated by the program and was bounded by the working range of the IGRT system. The 6D phantom simulated the compound 6D setup error of a patient by adopting these six figures on the phantom simultaneously. After taking the two stereoscopic images of the 6D phantom followed by software calculation, the 6D detection errors of ΔE=E′−ΔN−E were obtained. They were all found to be bounded within ±1mmand±1°. From Table 3, the mean and standard deviation of the 50 data for each dimension were given by μ±σ in the ΔE block. The significance p‐value for VERT and PITCH were less than 0.05, meaning that they could take that mean value. Other p‐values were greater than 0.05, meaning that the corresponding mean values were statistically zero at the 95% confidence level.

Every set of 6D ΔE components involved one resultant translational detection error and one resultant rotational detection error. They were the absolute, actual, scalar, spatial error magnitudes. In this study the mean of each of these errors (N=50) was 0.66 mm and 0.63°, respectively. The maximum was 1.22 mm and 1.33°, respectively. It is obvious that the resultants could be greater than 1 mm or 1°, though the individual components were all bounded by ±1mmand±1°.

#### A.3 Correction error ΔC

After robotic couch correction based on the 6D detected offsets E', a second detection was made to verify the correction efficacy. Since the detection efficacy of the IGRT system was established before, the detected 6D correction errors ΔC were directly obtained. They were all found to be bounded within ±1mmand±1°. From Table 3, the mean and standard deviation of the 50 data for each dimension were given by μ±σ in the ΔC block. As the detected shift vector E’ had already included AN, and the correction vector was exactly E', it is clear that ΔN shall not be subtracted from ΔC. The ΔC components were directly obtained from the postcorrection detection. From Table 3 it was found that the μ±σ values for the detection error and correction error were similar. As detection came first, it could be claimed that the correction errors were relatively low as compared with the detection errors by statistical finding (see Discussion section A below).

For resultant translational and rotational correction errors, the mean (N=50) was 0.50 mm and 0.54°, respectively. The maximum was 1.26 mm and 1.14°, respectively, as shown in Table 3.

### B. Scatter plots and correlations

Although the detection errors and the correction errors were found to be bounded (in Results section A), any trends of the data points should also be explored. In this study, it is interesting to investigate whether a component of ΔE would be dependent on the magnitude of the corresponding component of E by plotting scatter plots of ΔE against E. The same should be investigated on ΔC against E. Since the resultant errors, translational or rotational, are the actual spatial sum error magnitudes in practice, they should also be included in the study. Each of the scatter plots carried a correlation coefficient (r) with its significance value (p). These have been tabulated in Table 3. The null hypothesis was that there was no linear correlation (r=0) among the 50 data points. The Pearson's correlation formula calculates (r) and (p) for each data group. A value of p<0.05 implies that the (r) value calculated is statistically significant and the null hypothesis is rejected (i.e., r≠0). If p>0.05, the null hypothesis cannot be rejected and (r) shall be regarded as zero, meaning that the 50 data points were not correlated at the 95% confidence level.

#### B.1 ΔE vs. E

**Figure 12:**
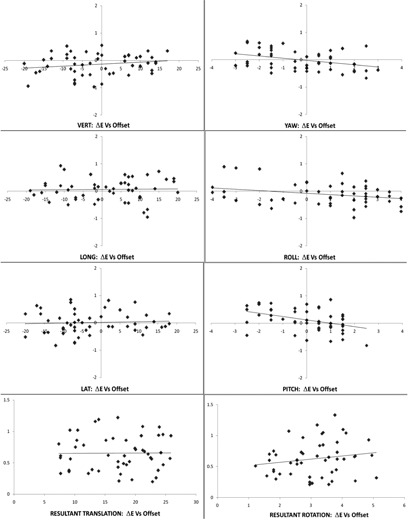
The PWH 2011 scatter plots of the detection error ΔE against the original 6D random offset E on the individual components and on the resultant values. The plots on the left column are for the translations in mm. The plots on the right column are for the rotations in degree. The individual linear regression lines are plotted as a function of the 50 data points.

According to the scatter plots of Fig. 12 and the data in Table 3, it was found that the three rotational components have significant weak negative correlation between ΔE and E with negative linear regression (LR) slopes (B). Their y‐intercept values (A) were close to zero. As ΔE=E′−ΔN−E, that means there were slight trends of proportional underdetection of E in the yaw, roll, and pitch components in both positive and negative directions of E. Such trends in vert., long., lat., and the two resultants were not statistically significant between ΔE and E. Detection error was found to be basically independent of its original offset value.

#### B.2 ΔC vs. E

**Figure 13:**
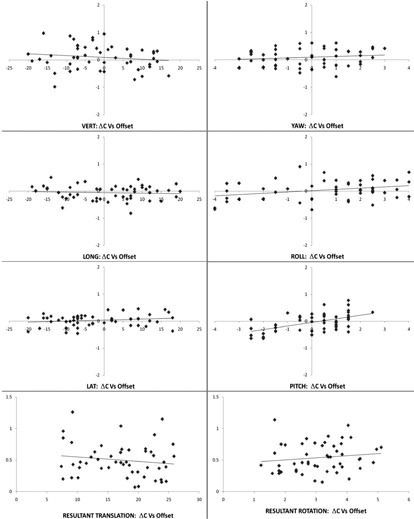
The PWH 2011 scatter plots of the correction error ΔC against the original 6D random offset E on the individual components and on the resultant values. The plots on the left column are for the translations in mm. The plots on the right column are for the rotations in degree. The individual linear regression lines are plotted as a function of the 50 data points.

According to the scatter plots of Fig. 13 and the data in Table 3, it was found that two rotational components, roll and pitch, have significant weak positive correlation between ΔC and E with positive LR slopes (B). Their y‐intercept values (A) were close to zero. As ΔC was the direct‐ read error after the correction, that means there were slight trends of proportional residual or undercorrection of E in the roll and pitch components in both positive and negative directions of E. This should be related to the slight underdetections of E mentioned above as supported by the matching of the opposite (B) slopes of the corresponding LR lines for ΔE and ΔC of roll and pitch, as shown in Table 3. Such trends in vert., long., lat., yaw, and the two resultants were not statistically significant between ΔC and E. Like the detection error, correction error was also found to be basically independent of its original offset value.

#### B.3 ΔC vs. AE

These scatter plots were done but not shown here. With the data in Table 3, it was found that all components except LAT of the correction errors showed significant weak to medium negative correlations with the corresponding detection errors. The (r) values of long, roll, and pitch reached 0.6 with negative sense. All the y‐intercept values (A) were close to zero. For overdetection events or components, overcorrection should follow since E’ would be greater than E and correction was based on E'. The phantom would be overshifted to the negative side proportionally and hence ΔC would be negative. The same argument applied to any underdetection case in which ΔE was negative and ΔC would be positive proportionally (Fig. 11). These general trends agreed with the experimental results and were found to be very logical. The resultant rotation plot of ΔC against ΔE showed a marginally significant correlation. The data mentioned in the Results sections B.1, B.2, and B.3 above mutually supported each other and hence confirmed the system characteristics that will be discussed in the Discussion section C below.

## III. DISCUSSION

### A. General experience on the IGRT and QA system

Although the ExacTrac IGRT and robotic system had been reviewed by different researchers before, this study involved a novel QA phantom cum program and took a completely different approach to evaluate the accuracy of the add‐on IGRT system. In this study, the detection errors and the correction errors were separated and analyzed individually. In fact they are of different natures but are coupled. The detection is based on X‐ray imaging and mathematical fusion. The correction uses the detection result and then applies mechanical servo‐tracking through the Infrared system monitoring. The detection performance established first becomes the support of the correction error evaluation. The detection errors and the correction errors were found to be similar in statistical parameters (Table 3). This claimed the high correction performance or negligible error exhibited by the infrared‐tracking robotic couch control mechanism. The results supported the studies of the Henry Ford group which stated that the infrared cameras could achieve 0.2 mm high resolution.^(9)^ This work confirmed that the overall error was due to the detection rather than correction.

For commissioning purpose, it is recommended to complete five QA worksheets over five days for the new IGRT machine. The basic standard of 1 mm and 1° accuracy of 6D components shall be met simultaneously by the new system for acceptance. The first year analytical results of 50 sets of 6D data (Tables 2 and 3 and 12, 13) shall become the benchmarks or the characteristics of the IGRT system and the 6D QA phantom, provided that the reference phantom CT image set remains unchanged.

The solid structures of the 6D phantom like the cube size, marker dots, marker rods, and part of the edges or faces could be changed to reduce the number of unnatural straight line images registered. Some other objects can be added internally to simulate human anatomical landmarks. It would be a problem if the phantom was raised from the supporting rod during the offset setting by the adjusting screws. This could be solved by drilling a thin hole at the center top of the phantom down to the Isocenter with a testing pin in it. A preset fine mark just appears on it would indicate a valid offset setting, a sunken mark would show the opposite.

Like the phantom in Fig. 3, this 6D phantom could be commercially produced by RT solution companies and become widely available. The performance and QA results among various centers with the same or different IGRT systems can then be compared systematically in the future. Compact single bi‐axes inclinometer sensor with digital output of pitch and roll angles simultaneously is available (Level Developments Ltd., Surrey, UK). The small device can be put onto the top of the 6D phantom and may further simplify the QA setup.

### B. The performance of the QA system and the IGRT system

It is essential to prove independently and comprehensively the ultimate performance of an equipment before the functionality of which could be established. Out of the 50 sets of raw and processed data presented, an ultimate figure about the overall performance of the IGRT system has to be stated. It is novel, fair, and objective to take the resultant translational and rotational correction errors in Table 3 to claim the overall accuracy of the system. They are 0.50±0.27mmand0.54±0.23°, respectively (mean±standard deviation) with N=50. Practically, the actual patient radiation treatment can proceed when all six detected 6D setup errors are within ±1mmand±1°. The system is so far stable and reliable, both in the QA results and in the patient setup. For the latter, care must be taken to check the oblique image fusions when the target is situated at the peripheral of the body where anatomical landmarks are scarce.

### C. System characteristics and accreditation

Besides the fulfillment of the basic requirement of the 1 mm and 1° 6D precision, the raw and derived data in Tables 2 and 3 and 12, 13 about ΔN, E, ΔE, ΔC are the characteristics of the IGRT system and the 6D QA system as a whole. Keeping the CT image set for the neutral configuration of the 6D phantom unchanged, these plots and the statistical figures should remain similar in their trends and values. International Task Group can be formed to collect these annual data from the 6D IGRT system users globally. With careful evaluation of the tables and the plots, accreditation can be awarded to those centers with reasonably high levels of IGRT QA standard. It is also interesting to investigate the performance differences between those added‐on IGRT systems and the built‐in ones, or between the ExacTrac X‐ray and the CBCT imaging system. The accuracy of the BrainLAB robotic couch against the Elekta HexaPOD mechanism can also be compared by this QA system.

### D. Clinical Implication

From this study, the accuracy of the 6D IGRT system is confirmed to be within 1 mm and 1° in every dimension, and the resultant translational (3D diagonal) setup error of a patient could be over 1 mm (Results sections A.2 and A.3). In PWH, the corresponding linac gantry sagging could be up to 0.5 mm as revealed by the Winston‐Lutz test currently. By these parameters of system accuracy and taking the root mean square of the errors, the CTV shall be expanded by a 1.5 mm margin in three translational dimensions to obtain the PTV for treatment planning. This is to avoid any spatial missing of the target in actual treatment. This 1.5 mm margin is reasonable and shall be sufficient to cover all the spatial uncertainties in the stereotactic treatment with this IGRT system. Detailed study about the practical spatial margin setting for treatment planning and the significance of the rotational errors shall be our further associated research topics.

## IV. CONCLUSIONS

The new 6D phantom cum QA program for IGRT system was successfully fabricated and applied to real settings in PWH. The BrainLAB ExacTrac X‐Ray and robotic couch IGRT system added onto Varian iX linac was commissioned and fully tested with the novel QA system developed. Random compound offsets were generated and simulated by the QA system on the IGRT system, which was proved to be sufficiently precise and is competent for stereotactic RT. Six‐dimensional ExacTrac detection errors and 6D robotic correction errors were found to be within 1 mm and 1° for the individual components. Offset magnitudes did not affect the IGRT system performance or accuracy. Final scalar resultant errors were analyzed in both translational and rotational senses, and these composite errors were found to be 0.50±0.27mmand0.54±0.23°, respectively. This end‐to‐end phantom study fully assured the accuracy and consistency of the IGRT system with static targets. As a consequence of this study, the target expansion by a 1.5 mm 3D margin from CTV to PTV is confirmed for this local IGRT system currently. With the expectation that the 6D phantom (patent pending) would later be commercially available, commissioning and routine QA for all local 6D IGRT system through this methodology should be promoted. Global cross‐comparisons and accreditations of this important RT modality should also be considered.

## Supporting information

Supplementary Material
